# Shared decision making and patients satisfaction with strabismus care—a pilot study

**DOI:** 10.1186/s12911-021-01469-y

**Published:** 2021-03-26

**Authors:** Ala Paduca, Oleg Arnaut, Eugeniu Beschieru, Per Olof Lundmark, Jan Richard Bruenech

**Affiliations:** 1 Faculty of Health and Social Science, South Eastern University Norway, Kongsberg, Norway; 2 Ophthalmology Department, Universitatea de Stat de Medicina si Farmacie “Nicolae Testemitanu″, Chişinău, Republic of Moldova; 3 Department of Human Physiology and Biophysics, Universitatea de Stat de Medicina si Farmacie “Nicolae Testemitanu”, Chişinău, Republic of Moldova; 4 Department of Surgery No.1 “N. Anestiadi”, Universitatea de Stat de Medicina si Farmacie “Nicolae Testemitanu”, Chişinău, Republic of Moldova

**Keywords:** Shared decision making, Patient satisfaction, Adult strabismus, Patient-centered care, Physician–patient communication

## Abstract

**Background:**

Strabismus is a complex disease that has various treatment approaches each with its own advantages and drawbacks. In this context, shared decisions making (SDM) is a communication process with the provider sharing all the relevant treatment alternatives, all the benefits, and risks of each procedure, while the patient shares all the preferences and values regarding his/her choices. In that way, SDM is a bidirectional process that goes beyond the typical informed consent. Therefore, it is known a little of the extent to which SDM influences the satisfaction with the treatment outcome along with strabismus patients. To study this correlation, an SDM-Q-9 questionnaire was provided within surgical consultations where treatment decisions were made; the SDM-Q-9 aims to assess the relationship between the post-operative patient’s satisfaction and their SMD score.

**Methods:**

The study is considered a prospective observational pilot study. Eligible patients were adult patients diagnosed with strabismus, who had multiple treatment options, were given at the right of choice without being driven into a physician’s preference*.* Ninety-three strabismus patients were asked to fill out the SDM-Q-9 questionnaire related to their perception of SDM during the entire period of strabismus treatment. After the treatment, patients were asked to rate their satisfaction level with the surgical outcome as excellent, good, fair, and poor. Descriptive statistics and the linear regression statistical tests (Spearman, Mann Whitney U, and Kriskal–Wallis) were used as analysis tools.

**Results:**

The average age of the participants was 24, where 50.6% were women. The mean SDM-Q-9 score among patients was 32 (IQR = 3). The postoperative patient satisfaction was rated as being excellent by 16 (17.2%) patients, good by 38 (40.9%), fair by 32 (34.4%), and poor by 7 patients (7.5%). Data analysis by linear regression statistical tests showed a positive correlation between the SDM-Q-9 score and the patient satisfaction related to the surgery outcome (B = 0.005, p < 0.001). Criteria in assessing patients’ satisfaction were age, gender, and strabismus type. A positive correlation between SDM and real satisfaction (r = 0.834, p < 0.01) was found with age, and no significant relationship was found while taking into consideration the responder’s gender and the strabismus type.

**Conclusions:**

Assessing patient satisfaction after choosing a treatment for strabismus method helped us evaluate the gaps in constructive dialogue that would lead to a positive outcome for both patient and clinician. The correlation between the SDM process and the patients’ satisfaction with surgery outcome, adjusted by age, has been established. These findings can serve as a springboard to further communicative improvements related to the SDM process and between patients and physicians, thereby consequently leading to patients’ satisfaction raise in strabismus care. The study underlines the importance of further analysis and validation of on-ground interactions among the adolescent and adult patients and the clinicians across the strabismus management trajectory. A multicentral study and its validation will follow.

**Supplementary Information:**

The online version contains supplementary material available at 10.1186/s12911-021-01469-y.

## Background

Adult strabismus is a complex, multi-dimensional disease, which has a serious impact on sightseeing, self-perception, self-esteem, and the social interactions of the patient [1]. Due to the esthetical disturbance of the disease to adolescents and adults, especially when considering people with longstanding, childhood-onset strabismus, the relating prejudice to eye deviation extends beyond social interactions. The complexity of decisions in these patients involves all aspects of strabismus management: evaluation, diagnosis, and treatment. In this context, the collaborative or shared decision is seen as a model for clinical practice [2] and was defined by Weston in 2001 as being one of the key components to patient-centered care [3]. Several treatment options, including optical correction, prism prescription, various surgical approaches, and botox injections are significant, each with the own advantages and disadvantages [4]. Therefore, the choice of therapy requires a careful examination, where the available scientific data, the experience of the clinician as well as the characteristics and preferences of the individual patient are weighed together upon the taken decision [5]. These factors may add stress to the patient, family, and the clinicians involved in the strabismus care. However, all surgical treatment options, including the option not to operate, would always have their possible positive and negative outcomes to make space for the patient's preferences. This patient-physician alliance results in empowering patients to develop their autonomy [6, 7] and in finding better healthcare choices.

The World Health Organization and UNICEF have highlighted the need to involve teenagers in their treatment decisions [8, 9]. Furthermore, earlier research performed by Wisdom et al. [10] outlined that teens are eager to exchange information with clinicians and they want to express their opinion and to have the autonomy to choose between treatment options whenever possible. A young person’s ability to consent depends more upon his understanding of the nature of the proposed treatment and its possible consequences [11]. Shaw [12] mentioned that there is no simple criterion or measure of the competence; the doctors’ skill lies in assessing the young patient’s competence in a particular context for a specific decision.

Gärtner et al. [13] in a literature search identified 16 existing patient profiles related to SDM. A general nine-point decision-making questionnaire is one of the most used tools to assess the extent to which doctors involve patients in the decision-making process. It consists of versions of the patient (SDM-Q-9) and the doctor (SDM-Q-Doc), which can be used to evaluate the patient's involvement in the decision-making process from two points of view [14, 15]. It is often used in various clinical situations, including primary [16] and specialized care as oncology [17], otorhinolaryngoglogy [18], mental health practice [19], vascular surgery [20], dialysis [21] multiple sclerosis [22] and even among patients’ family members [23]. It has been translated into many languages since 2009, including Romanian [14, 24].

Several available publications in the ophthalmological field directed towards managing patients with cataract, diabetic retinopathy, glaucoma, age-related macular degeneration, and exotropia in providing a qualitative analysis by using standard interviews with healthcare workers and/or patients [25–29] and support the need for more patient involvement in shared making decision process. So far, none of these studies aimed to estimate the outcome of the SDM on the patients’ satisfaction with the final/post-op result.

Although, SDM has become a priority of health policy in many European countries in the past two decades [30] there are no studies on the involvement of patients in taking medical decisions in Western Europe and the Republic of Moldova. Mainly, the promotion of shared decision-making in strabismus treatment is a field that has not been researched yet. Finding an association between the SDM process and the patients’ satisfaction with the outcome would help us work towards optimizing the SDM process and facilitate it towards a better comprehension for the patient- that way acquiring an optimized patient-centered care process.

We aimed to evaluate the impact of the shared decision-making process in adult strabismus on the patient’s postoperative satisfaction from the patients’ perspective. As the main tool, we have used the SDM-Q-9 questionnaire and confronted the score with a Face-Q scale: to compare the postoperative satisfaction and the decision-making score. The outcome had to help us improve the quality of the surgical consultations in the process of SDM and help both sides move towards taking an optimal decision. We wanted to clarify whether in the process of SDM the patients were satisfied with the amount and clarity of the options and outcomes and it led to a higher satisfaction rate vs. having the physician offer the best option based on.

## Methods

### Study design and context

We conducted a prospective observational pilot study among teens (15–17 years old) and adults over 18 years old undergoing strabismus surgery. This study was implemented in the Republic of Moldova at the Republican Clinical Hospital “Timofei Mosneaga” and Children Hospital “Emilian Cotaga” from January 2017 to December 2019, was carried out following the principles of the Helsinki Declaration and approved by The Ethics Committee of the State University of Medicine and Pharmacy” Nicolae Testemitanu”.

### Participant recruitment and eligibility

Among the participants were 93 adult patients with manifest strabismus who needed a surgical correction and in whom more than one technique was advised (eye muscle recession or resection by symmetric surgery, asymmetric surgery, or adjustable sutures). The patients were enrolled in this study if they met the following criteria: (1) 15 years and older at the time of surgery, (2) a confirmed diagnosis of manifest concomitant strabismus through orthoptic examination, (3) candidates for surgical correction of strabismus, and (4) Romanian communicative skills and written agreement of patients aged 18 and up, and from the teenagers' parents (for the teenagers between 15–17). Therefore the written informed consent was obtained from all participants prior to their enrollment in the study. The exclusion criteria were as follows: (1) severe cognitive impairment and (2) study involvement disapproval. The patients were informed about the study by their healthcare provider. They were thoroughly informed and assured that refusing to participate would not affect their treatment in any way.

### Procedure

Before the study, basic demographic data on age, gender, and type of strabismus were collected. All patients underwent a detailed ocular examination and an orthoptic evaluation before undergoing strabismus surgery.

After the final consultation, the patients completed the translated Romanian version of a validated patient-reported measure of shared decision making (the 9-item Shared Decision Making Questionnaire, SDM-Q-9) questionnaire. In this previously approved questionnaire, the SDM level was assessed subjectively by evaluating the nine phases of the decision-making process from the patient's perspective on a 6-point Likert scale ranging from 0 (not at all) to 5 (fully applicable) [13] (see Additional file 1: Appendix SDM-Q-9 questionnaire). Romanian version had good internal consistency with a 0.96 Cronbach α coefficient [31].

**Fig. 1:**
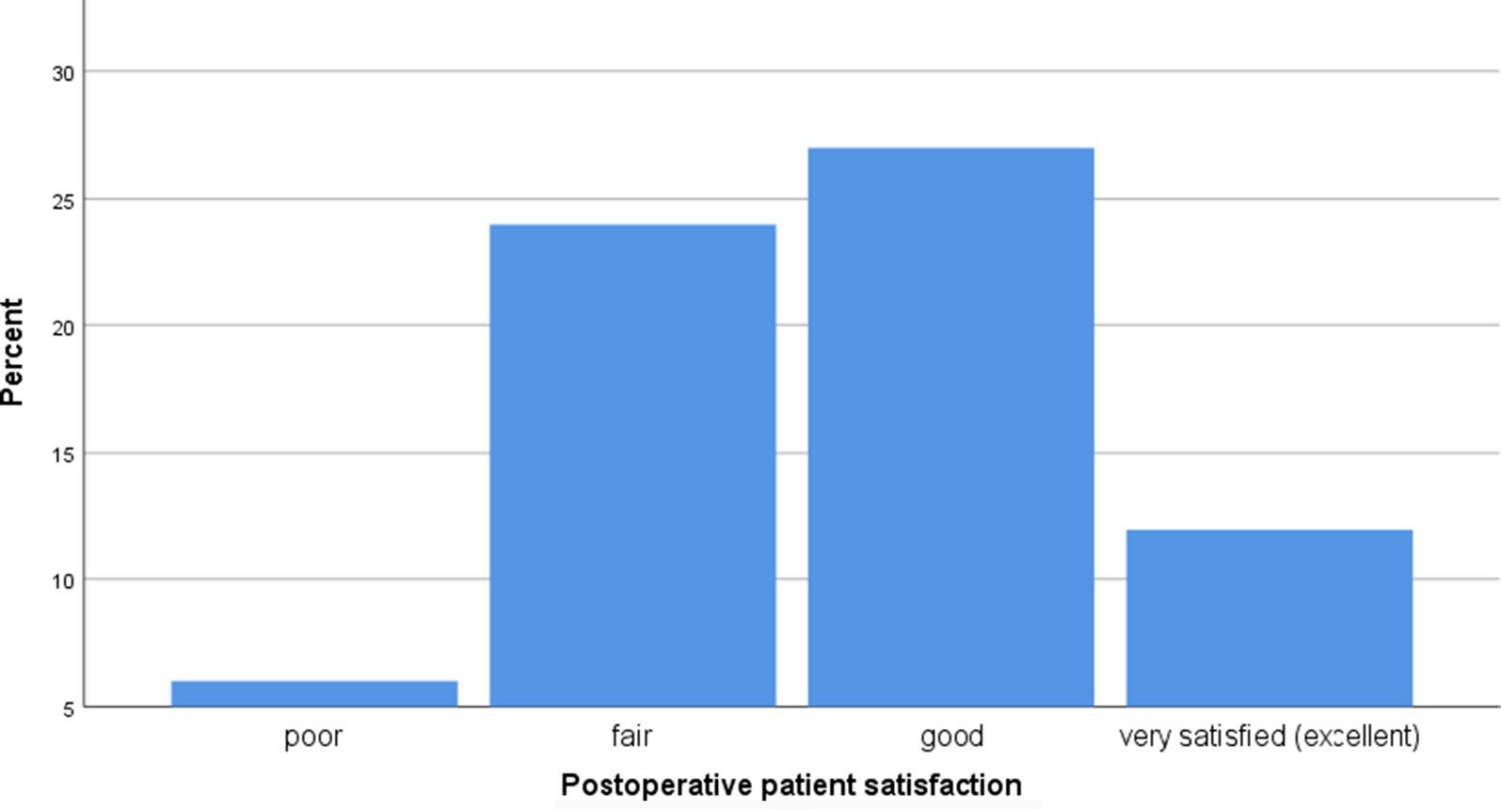
Postoperative patient satisfaction Face -Q-4 pont scale

10–12 weeks after the surgery the patients were asked to rate their satisfaction with the surgical outcome (POS) as excellent, good, fair, and poor using the Face-Q scale (a 4-point scale) [32] Fig. 1.

An excellent rating meant that the patients did not notice any eye deviation after the surgery interval. A good rating meant they have noticed slight strabismus occasionally. A fair (satisfactory) rating represented a slight degree of eye deviation present all the time. Finally, a poor rating represented their dissatisfaction with the residual amount of deviation.

### Statistical analyses

The SDM-Q-9 scores ranged between 0 and 45 (0 = no SDM behavior; 45 = ideal SDM behavior) [16]. A detailed analysis using the Statistical Package for Social Sciences, Version 26 (IBM SPSS Inc.) was applied. For continuous data, descriptive statistics were expressed as average /95% CI for median and interquartile range (IQR). For categorical data, descriptive statistics were expressed as absolute frequencies, percentages, and 95% CI. Potential relationships were visualized by a bubble plot diagram. Tests for data normality, transformation, and multiple linear regression analysis were conducted to evaluate the relationship among variables. Therefore, the statistical significance for all tests was defined as p < 0.05 level.

## Results

### Study population and baseline characteristics

Ninety-three patients aged 15 to 88 years old took part in the study with average age—24 years. Female represented 52.7% (49 patients), male—47.3% (44 patients). 42 (45.2%) patients were diagnosed with esotropia and 51 (54.8%) with exotropia.

Age distribution in accordance to Strabismus Type and gender is presented in Table 1.

**Table 1:** Age distribution of the patients

	Strabismus type			
	Esotropia		Exotropia	
	Female	Male	Female	Male
Age, years				
Median (95% CI)	23 (19–26)	23 (23–28)	27 (24–34)	25 (23–33)
Percentile 25	17	20	23	19
Percentile 75	30	24	41	33
IQR	13	4	17	14

### Patients’ views on involvement in strabismus treatment decision-making

The average SDM-Q-9 score among all patients was 32 (IQR = 3) and they fluctuated between 25 and 36. The mean SDM-Q-9 score assigned by esotropic female patients was 32 (IQR = 2) and a score of 33 (IQR = 2) was given by males. The exotropia patients conferred a similar median SDM-Q-9 score of 31 (IQR = 4), either females or males. Table 2.

**Table 2:** SDM-Q-9 score in accordance with Strabismus type and patient gender

	Strabismus type			
	Esotropia		Exotropia	
	Female	Male	Female	Male
SDM score				
Median (95% CI)	32 (32–34)	33 (32–34)	31 (30–33)	31 (29–32)
Percentile 25	31	32	29	29
Percentile 75	33	34	33	33
IQR	2	2	4	4

The lowest rated was item 2 (patients’ involvement) with a score of 3 given by 48 responders (51.6%) (95% CI 41.5–61.6), item 5 (patients' information) was graded with score 2 by 32 responders (34.4%), (95% CI 25.3–44.4), item 6 (patients’ preference), graded with score 3 by 77 patients (82.8%) (95% CI 74.2–89.4), 65 patients (69.9%) rated item 7 (weighing options) a score of 3 (95% CI 60.1–78.5) and 50 (53.8%) patients with same score rated item 8 (shared decision) (95% CI 43.6- 63.7) Table 3.

**Table 3:** SDM-Q-9 descriptive statistic by Items

Item	Score	Nr of patients	% (CI 95%)
SDMQ1	3	3	3.2 (0.9–8.4)
	4	65	69.9 (60.1–78.5)
	5	25	26.9 (18.7–36.5)
SDMQ2	2	4	4.3 (1.5–9.9)
	3	48	51.6 (41.5–61.6)
	4	41	44.1 (34.3–54.2)
SDMQ3	3	19	20.4 (13.2–29.5)
	4	67	72.0 (62.4–80.4)
	5	7	7.5 (3.4–14.2)
SDMQ4	2	1	1.1 (0.1–4.9)
	3	15	16.1 (9.7–24.6)
	4	74	79.6 (70.5–86.8)
	5	3	3.2 (0.9–8.4)
SDMQ5	1	2	2.2 (0.4–6.7)
	2	32	34.4 (25.3–44.4)
	3	44	47.3 (37.4–57.4)
	4	15	16.1 (9.7–24.6)
SDMQ6	1	1	1.1 (0.1–4.9)
	2	7	7.5 (3.4–14.2)
	3	77	82.8 (74.2–89.4)
	4	8	8.6 (4.2–15.6)
SDMQ7	2	19	20.4 (13.2–29.5)
	3	65	69.9 (60.1–78.5)
	4	9	9.7 (4.9–16.9)
SDMQ8	2	8	8.6 (4.2–15.6)
	3	50	53.8 (43.6–63.7)
	4	35	37.6 (28.3–47.7)
SDMQ9	3	2	2.2 (0.4–6.7)
	4	60	64.5 (54.5–73.7)
	5	31	33.3 (24.4–43.3)

### Correlation between the SDM score and postoperative patient satisfaction

The postoperative patient satisfaction was ranked as excellent by 16 (17.2%) patients, good by 38 (40.9%), fair by 32 (34.4%), and poor by 7 patients (7.5%). The POS score in accordance with strabismus type and gender is presented in Table 4.

**Table 4:** Patients satisfaction desciptive statistic

POS score		Strabismus type			
		Esotropia		Esotropia	
		Female	Male	Female	Male
Poor	n	0	0	3	4
	% (CI 95%)	0 (–)	0 (–)	11.1 (3.2–26.8)	16.7 (5.9–34.9)
Fair	n	7	3	10	12
	% (CI 95%)	31.8 (15.5–52.6)	15 (4.4–34.9)	37 (20.9–55.8)	50 (31–69)
Good	n	12	11	10	5
	% (CI 95%)	54.5 (34.3–73.7)	55 (33.8–74.9)	37 (20.9–55.8)	20.8 (8.4–39.4)
Excellent	n	3	6	4	3
	% (CI 95%)	13.6 (4–32.1)	30 (13.6–51.7)	14.8 (5.2–31.5)	12.5 (3.6–29.7)

The interrelation between SDM-Q-9 score and patients’ satisfaction, following to bubble plot, had to be adjusted to other potential covariates like age, gender, and strabismus types.

The bubble plot analysis of the relationship between the parameters included in the research such as patients' age, strabismus type, SDM -Q-9 score, and patients’ post-surgical satisfaction was performed.

**Fig. 2:**
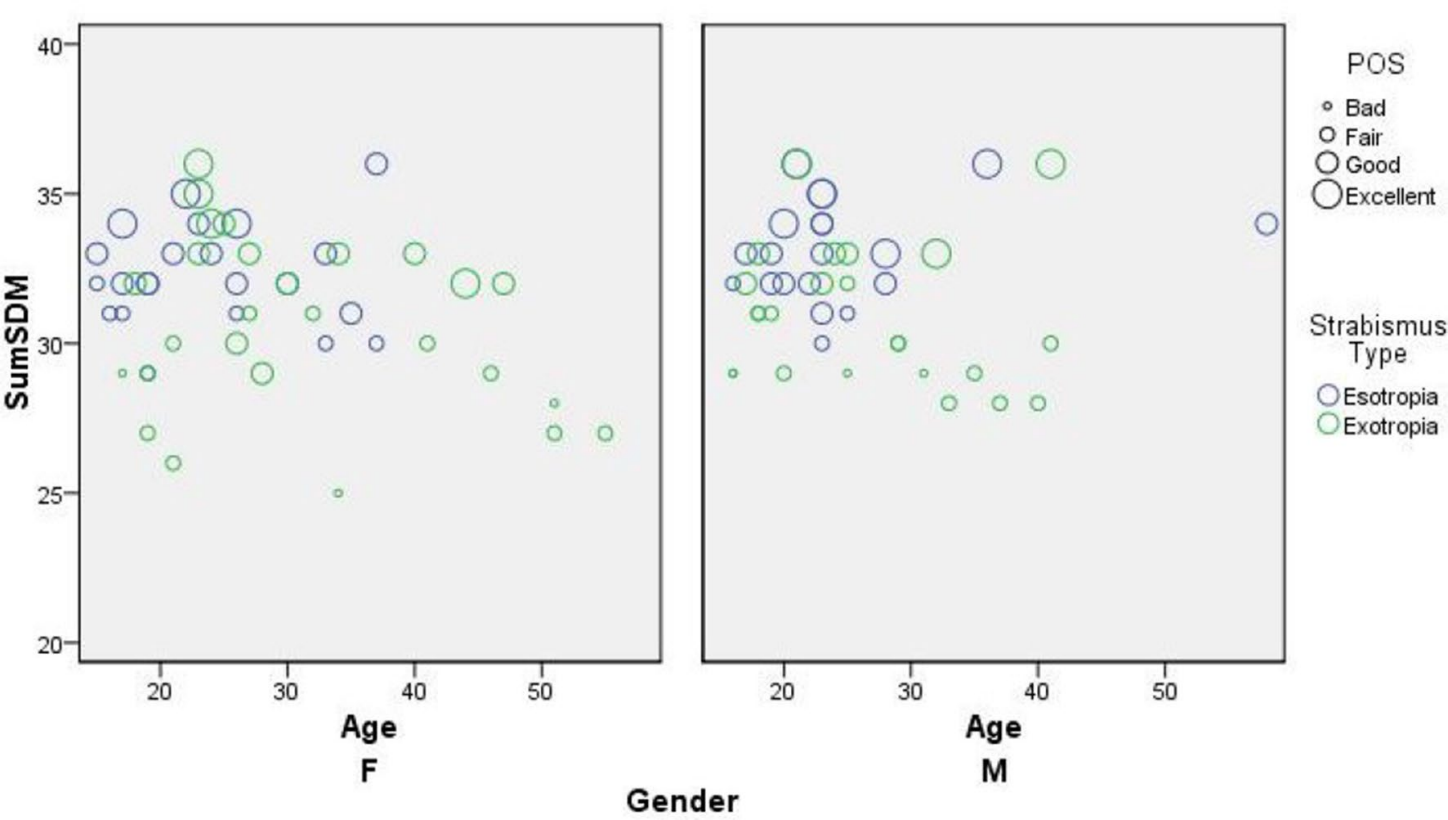
Buble plot analysis of relationship between variables

Figure 2 shows uncovered gender- and age-based differences such that men under 35 were the undermost satisfied group with SDM. Patients with exotropia, especially men, reported lower SDM and POS scores compared to esotropic patients. Furthermore, there is a positive relationship between the SDM score and the patient satisfaction level with surgery outcome (Fig. 2).

**Fig. 3:**
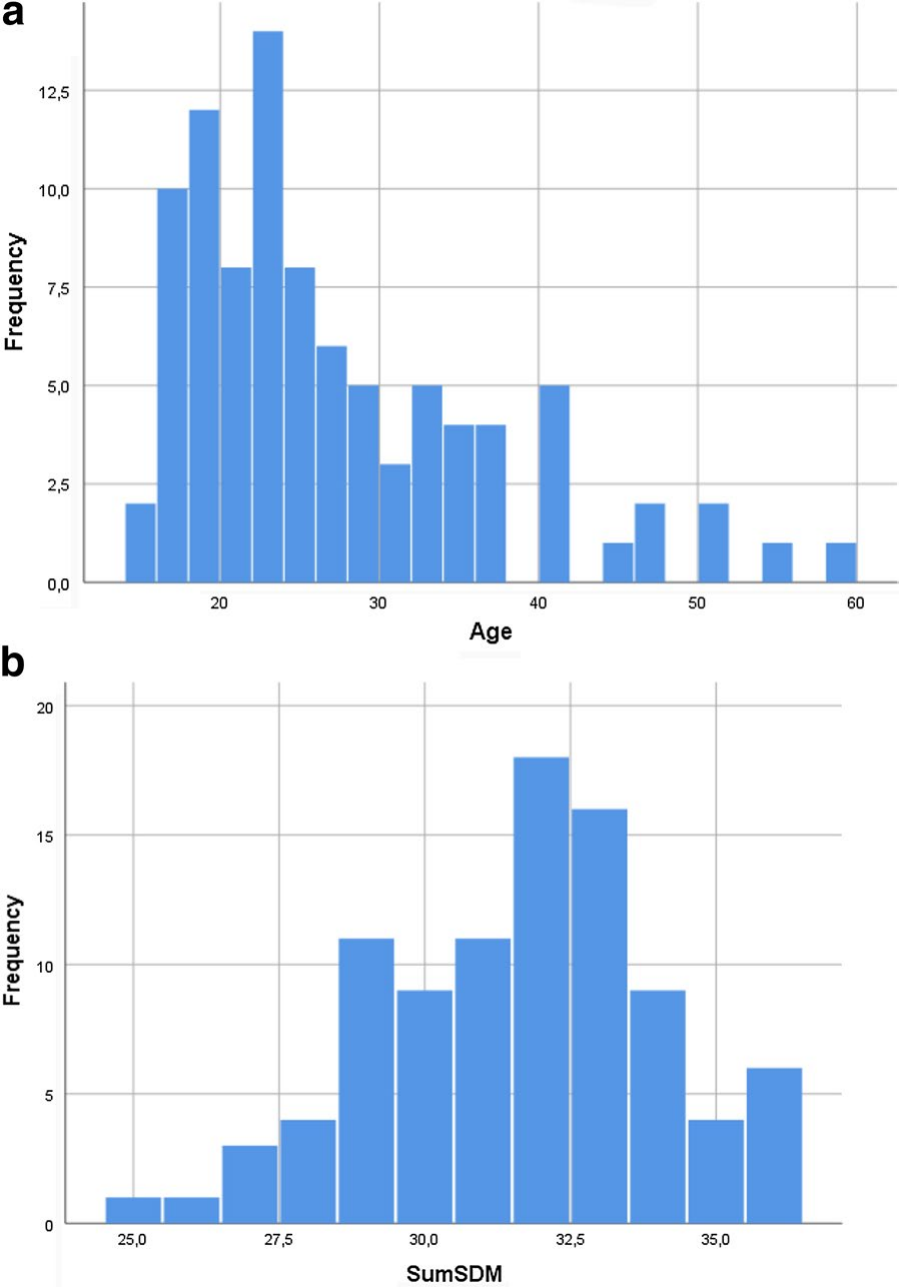
The histograms for normality distribution analysis for Age (**a**) and SDM score (**b**) variables. *Note* **a** Nr-93. Mean age -26.86 (SD = 9.62). **b** Nr-93. Mean SumSDM- 31.61 (SD = 2.42)

The multivariate analysis (linear regression) was chosen to comprehend the relationship between the SDM-Q-9 score and the Patient Satisfaction with surgery outcome level. Taking into account the abnormal distribution for both continuos variables Age (Skewness = 1.216, error standard = 0.250; Kurtosis = 1.107, error standard = 0.495; Shapiro–Wilk test = 0.888, df = 93, p < 0.001) and SDM -Q-9 score (Skewness = − 0.279, error standard = 0.250; Kurtosis = − 0.198, error standard = 0.495; Shapiro–Wilk test = 0.971, df = 93, p = 0.038) after histogram analysis (Fig. 3) the data restoring procedure was performed.

There were 2 new variables as results – Agenorm (Agenorm = 1/Age) and SumSDMnorm (SumSDMnorm = SumSDM^2), Shapiro–Wilk test being estimated as 0.978 df = 93 p = 0.109 for Agenorm and Shapiro–Wilk test = 0.974, df = 93, p = 0.062 for SumSDMnorm. Thus, continuous covariates normal distribution was respected.

The patients’ satisfaction score was considered as a dependent variable, Agenorm, SumSDMnorm, Gender, and Diagnosis as variables for the potential model.

The final model had the following characteristics: the model showed the abilities to predict the satisfaction value (F = 103.004, p < 0.001); the correlation between predicted and real results was estimated as 0.834, determination coefficient being 0.689 (68.9%); about 70% of satisfaction dispersion was determined by the variables from the equation. The sum SDM norm determination effect was the important one having a value of 0.679, Agenorm represented only 0.01.

**Fig. 4:**
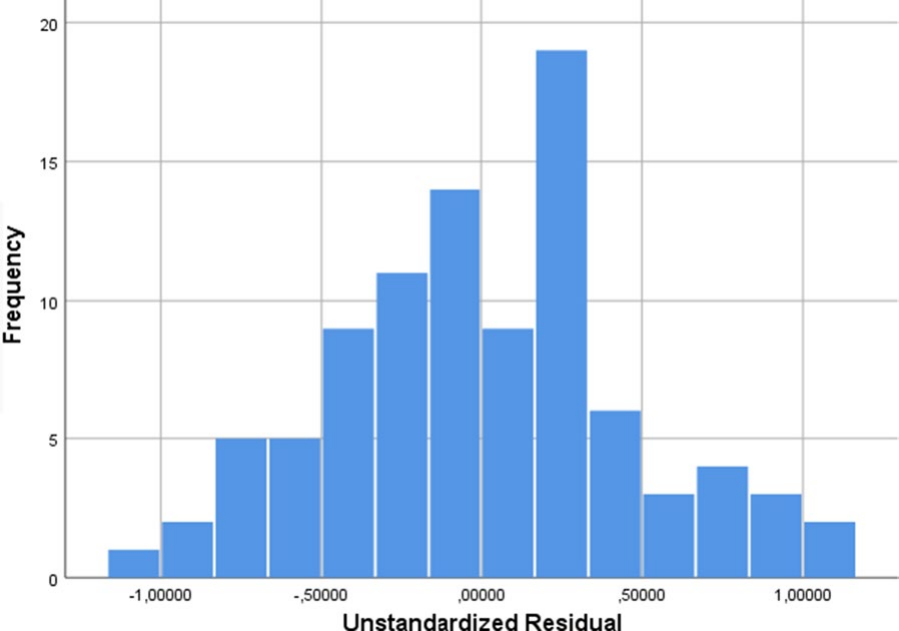
The histogram for residuals distribution. *Note* Nr -93. Mean 3.53E-16 (SD = .47)

The model included a constant (B = − 1.175, 95% CI − 2.417, − 1.013) and two transformed continuous variables, Agenorm (B = − 8028, 95% CI − 15.952, − 0.105) and SumSDMnorm (B = 0.005, 95% CI 0.004, 0.005), the SumSDMnorm effect estimated after coefficient standardization being higher in comparison with Agenorm (0.84 versus − 0.118). Collinearity testing showed no interaction between covariates (Tolerance = 0.985, VIF = 1.015) (Table 5). Type of strabismus and gender did not show any relevance. Residuals were normally distributed (Fig. 4), Shapiro–Wilk test = 0.993, *df* = 93, *p* = 0.906.

**Table 5:** Collinearity test among variables

	Coefficients, model								
	Unstandardized coefficients		Standardized coefficients	t	Sig.	95.0% confidence Interval for B		Collinearity statistics	
	B	Std. error	Beta			Lower bound	Upper bound	Tolerance	VIF
(Constant)	− 1.715	0.353		− 4.852	0.000	− 2.417	− 1.013		
Agenorm	− 8.028	3.988	− 0.118	− 2.013	0.047	− 15.952	− 0.105	0.985	1.015
SumSDMnorm	0.005	0	0.84	14.35	0.000	0.004	0.005	0.985	1.015

According to the coefficients, the Age norm had a negative effect with large confidence intervals; the sum SDM norm had a positive effect with narrow confidence intervals.

## Discussion

Shared decision making (SDM) comprises three main elements: the exchange of information (personal and medical) between the patient and the doctor, the discussion of the diagnosis and the treatment options, and the building of consensus [33, 34].

### Patients’ views on involvement in strabismus decision-making treatment

In this study we wanted to outline the patients’ perception on the treatment decisions about strabismus shared by both the doctors and the patients.

We have identified five key concerns among our patients on the SDM process. This research pointed relatively low scores to SDM-Q-9 items 2 (patients’ involvement), 5 (patients' information), 6 (patients’ preference), 7 (weighing options), and 8 (shared decision). This means that the physician has not always rated adequately or met the patients’ informational needs and did not actively invite patients to share their goals, expectations, and concerns.

Compared to patients from the other healthcare institutions who had an active role in strabismus decision-making treatment our patients rated a 71% score on involvement, compared to higher results observed in breast cancer patients (78–83%) [35, 36], 93.3% in patients with multiple sclerosis [22] and 95% in primary and secondary care [33], while Nakashima et al. (2012) reported a similar score with our 70% [37].

One of the main components of SDM is to determine patient's values and preferences on different treatment options [38, 39]. This study exhibited that identifying and considering patients' preferences is not a common practice yet according to item 6 score. The concept of SDM implies both clinicians and patients work together in order to select the best option using scientific evidence by applying providers’ clinical experience and patients’ preferences [40]. Therefore, patients' preferences are totally different from the doctor's options. Consequently, not all the doctors are ready to discuss patients’ values and needs; otherwise, some feel like their clinical experience will be compromised if the patient disagrees with their recommendations. If patients disagree with the treatment recommendations, this may be due to a mismatch between their preferences and the perception of these preferences by doctors. Benbassat et al. [41] (1998) revealed that doctors' conclusions about patients' values and preferences are often inaccurate, even for the doctors with more clinical experience and a longer doctor-patient relationship.

It is not astonishing, that Tamirisa et al. [42] concluded that many patients got serious problems during SDM [30]. A study performed by Sharma et al. [43] on a sample of 60 patients revealed that only 30% of the patients were aware of the strabismus pathology and the treatment alternatives. This may also be the reason why the patients in our study have rated the items 7 (weighing options) so poorly—there was no clear balance towards one of the existing treatment methods. The obtained results indicated that some patients believe that their doctors had not provided the amount of information that they wanted to receive. This raises a question about the possible lack of perception of the way a shared decision-making procedure should be conducted.

Another concern revealed in this study was related to the fact that some patients felt excluded from the treatment decision making process. Similar to the data reported by Lecouturier et al. [25], our patients expressed the desire to be involved in medical decisions that concern them. This preference seems to be stronger than their doctors’ willingness to share decisions with them according to the rated score of item 8. Our study results clearly show that the information providing about existing treatment options is clearly explained, and patients need this information to be able to participate in SDM.

On contrary, a quite high score was given to item 9 meaning that finally an agreement about further treatment procedures was achieved. This led us to the conclusion that probably some patients are still more confident in their doctor knowledge, recommendation and experience. Otherwise, some are not eager to share the responsibility for decisions and leave it to surgeons. SDM is a time-consuming process and this fact has also been mentioned by other authors [25, 38]. Some patients need more time to assimilate and become aware of the information received from the doctor, especially adolescent patients. Recent researches suggest that minors up to 12 years old are capable of being involved in decision making [44, 45]. Studies on the role of teenagers and their involvement in the decision-making process usually report parents' points of view instead of teenagers [25]. Even if most studies revealed that adolescents desire active involvement in their treatment decisions, disagreement remains over their capacity to do so from the perspective of parents, providers, and the healthcare system. Instead of, our study revealed teens seemed relatively satisfied with the SDM process, showing a score nearly similar to that reported by the young adults. They are eager to discuss their problem, to have an interactive process rather than co-operating just to “hear” a quick diagnosis and follow the doctor's recommendations.

### Patient satisfaction with surgery outcomes and the relation with SDM score

Improving the lives of patients with longstanding strabismus requires a bivalent collaboration: patients should understand their choices and the possible outcomes of those choices, and providers should understand the needs and expectations of their patients—good communication between them is the key to achieving the best result. In this study the participants reported an overall positive experience with their care: 58.1% were satisfied or very satisfied with the results of their surgery, and 7.5% were unsatisfied. Having been compared to other publications, a higher result of overall 90% of parents' satisfaction with strabismus treatment rated as "good" or "very good" was reported by Mruthyunjaya et al. [46] and Kaszli et al. [47].

We have to point out the existence of disease-specific and non–disease-specific factors that may influence patients’ feedback [48] – these captures big attention from researchers lately. In this study was obvious that the level of effective decision-making influenced the level of satisfaction with treatment outcome. Communication that provides the evidence-based, weighted level of assistance to patients in their decision making appears to be an important non-desease- specific factor that influence the SDM process and the patients’satisfation with received healthcare.

Furthermore, in our study, no significant difference was observed in reported satisfaction levels among males and females and among esotropic and exotropic patients. Similar results were obtained by Kaszli et al. [47] in a study on patients undergoing strabismus surgery no significant difference in patient satisfaction was found. On the other hand, Burke et al. [49] found that people with an esotropic strabismus recorded significantly greater appreciation of the effects of surgery than those with an exotropic strabismus**.** However, comparing both surgeons' and patients' opinions, Burgos-Blasco et al. [50] studied the surgeons’ point of view and concluded that endotrophic is the most satisfactory surgery for the patient, followed by exotropia, vertical strabismus, and traumatic paralysis of the 4th cranial nerve.

Our results suggest that the higher the SDM score, which means higher patient involvement in their healthcare process, the higher is their satisfaction with the provided care. In our research the interrelation between the SDM process and the patient satisfaction with surgery outcome, adjusted for age, has been established, the predictive value having a powerful association with the real value. SDM offers a process that can help both the physician and the patient move beyond passive informed consent to a more collaborative, patient-centered experience.

This study identifies the communication gaps from the patients’ perspective, helps us improve discussions in strabismus management, and illustrates the value of understanding how treatment decisions about strabismus are currently made upon the final patients’ satisfaction.

### Strengths and limitations of this study

This study was an attempt to qualitatively assess the patients’ experience of treatment decision making for strabismus in the Republic of Moldova. The study evaluated the correlation between the patients’ involvement in the collaborative decision process and the satisfaction with management outcome.

This study has some limits. First, the small number of participants limits a wide generalization to all population with strabismus. A more representative sample of patients may bring out different results than the ones we received. The described model needs correction by efficient variables enrolment because of reduced determination coefficient (about 70%), another 30% of variance being unexplained. The potential determinants for patients’ satisfaction, besides the age and SDM, could be the education level, sociodemographic and clinical status, etc. [51, 52]. Also, the elaborated model for satisfaction prediction needs validation using an independent sample. The data transformation (age) to meet conditions for linear regression represents another limitation.

However, it has just theoretical application and can be corrected in large studies when all continuous variables will have a normal distribution. Secondly, one of the parents were always present during consultations and shared decision making evaluation process, which may influence the adolescents’ answers – so they should probably be assessed in a separate study or as a separate group.

## Implications

Taking into account the results and listed limitations we plan to extend our research. The number of patients will be increased by performing a multicentral study. Other potential factors (education level, socioeconomic situation, strabismus type, surgery technique, etc.) that could affect the relationship between the SDM process and patient satisfaction also will be taken into account. We expect the net effect estimation of SDM adjusted for these predictors, explained variance for satisfaction will be optimized (more than 80%). Finally, the results will be validated using an independent sample. The final goal is to estimate the SDM benefits in multivariate/multifactorial “space” and to implement this procedure in routine practice.

## Conclusion

Assessing patient satisfaction after choosing a treatment for strabismus method helped us evaluate the gaps in constructive dialogue that would lead to a positive outcome for both patient and clinician. The correlation between the SDM process and the patients’ satisfaction with surgery outcome, adjusted by age, has been established. These findings can serve as a springboard to further communicative improvements related to the SDM process and between patients and physicians, thereby consequently leading to patients’ satisfaction raise in strabismus care. The study underlines the importance of further analysis and validation of on-ground interactions among the adolescent and adult patients and the clinicians across the strabismus management trajectory. A multicentral study and its validation will follow.

## Supplementary Information

**Additional file 1.** The 9-item Shared Decision Making Questionnaire (SDM-Q-9).

## Data Availability

The dataset generated and analyzed in this study are not publicly available yet because it is a part of the PhD work in progress. Data information is available from the corresponding author. Note that the questionnaires are in Romanian.

## Abbreviations

SDM: Shared decision making
SDM-Q-9: 9-Item Shared Decision Making Questionnaire
POS: Postoperative patient satisfaction
